# Gene-Based Rare Allele Analysis Identified a Risk Gene of Alzheimer’s Disease

**DOI:** 10.1371/journal.pone.0107983

**Published:** 2014-10-20

**Authors:** Jong Hun Kim, Pamela Song, Hyunsun Lim, Jae-Hyung Lee, Jun Hong Lee, Sun Ah Park, for the Alzheimer’s Disease Neuroimaging Initiative

**Affiliations:** 1 Department of Neurology, Dementia Center, Stroke Center, Ilsan hospital, National Health Insurance Service, Goyang-shi, South Korea; 2 Department of Neurology, Inje University Ilsan Paik Hospital, Goyang-shi, South Korea; 3 Clinical Research Management Team, Ilsan hospital, National Health Insurance Service, Goyang-shi, South Korea; 4 Department of Life and Nanopharmaceutical Sciences and Department of Maxillofacial Biomedical Engineering, School of Dentistry, Kyung Hee University, Seoul, South Korea; 5 Department of Neurology, Soonchunhyang University Bucheon Hospital, Bucheon-shi, South Korea; University of Leipzig, Germany

## Abstract

Alzheimer’s disease (AD) has a strong propensity to run in families. However, the known risk genes excluding *APOE* are not clinically useful. In various complex diseases, gene studies have targeted rare alleles for unsolved heritability. Our study aims to elucidate previously unknown risk genes for AD by targeting rare alleles. We used data from five publicly available genetic studies from the Alzheimer’s Disease Neuroimaging Initiative (ADNI) and the database of Genotypes and Phenotypes (dbGaP). A total of 4,171 cases and 9,358 controls were included. The genotype information of rare alleles was imputed using 1,000 genomes. We performed gene-based analysis of rare alleles (minor allele frequency≤3%). The genome-wide significance level was defined as meta *P*<1.8×10^–6^ (0.05/number of genes in human genome = 0.05/28,517). *ZNF628*, which is located at chromosome 19q13.42, showed a genome-wide significant association with AD. The association of *ZNF628* with AD was not dependent on *APOE* ε4. *APOE* and *TREM2* were also significantly associated with AD, although not at genome-wide significance levels. Other genes identified by targeting common alleles could not be replicated in our gene-based rare allele analysis. We identified that rare variants in *ZNF628* are associated with AD. The protein encoded by *ZNF628* is known as a transcription factor. Furthermore, the associations of *APOE* and *TREM2* with AD were highly significant, even in gene-based rare allele analysis, which implies that further deep sequencing of these genes is required in AD heritability studies.

## Introduction

Alzheimer’s disease (AD) is a leading cause of dementia and is known to have high heritability (as high as 60–80%) [1], [2]. Genome-wide association studies (GWAS) have identified several risk genes for AD such as *ABCA7, BIN1, CD33, CD2AP, CLU, CR1, EPHA1, MS4A6A/MS4A4E*, and *PICALM* [3]–[7]. The known risk genes for AD explain only 30% of heritability [8], [9]. Aside from *APOE* ε4, reported risk genes have low clinical significance because of their small effect sizes [7]. The common variant hypothesis posited common diseases are attributed to common variants and this hypothesis is base concept for GWAS [10], [11]. However, similar to other common diseases, the heritability of AD cannot be fully explained by common alleles [12].

There are growing reports regarding rare variants related to complex diseases [13]–[17]. Contrary to the common variant hypothesis, variants with low frequency could be primary causes for common diseases, according to the rare variant hypothesis [11], [18]. The rationale of the rare variant hypothesis is that allele variants with low frequencies have a higher probability of functional significance [12]. A large scale exome sequencing study has indicated that 95.7% SNPs with functional importance are rare variants [19]. Additionally, the number of variants with loss of function showed an inverse correlation with MAF [20], [21]. Considering their functional significance, rare variants may have large effect sizes. Recently, rare alleles in *TREM2*, *APP,* and *PLD3* have been reported to have association with AD [22]–[24]. Thus, the identification of more risk or protective rare alleles associated with AD is required.

Although rare alleles are promising targets for genetic association studies of complex diseases, the analyses of rare alleles remains challenging. For example, very large sample sizes are required to detect rare alleles that have modest effect sizes [19]. Deep sequencing of large samples is too expensive for typical researchers to perform. The mutational loads within the same genes, regions, or pathways can be alternative approach [13], [25]. However, a large number of candidate rare alleles within specific regions are more difficult to obtain and interpret, than genotyping of a few loci.

Improvement of imputation methods has allowed accurate inference of rare alleles [26]. According to 1000 genomes study [20], the mean squared Pearson correlation coefficients (*R* ^2^) between rare SNPs (MAF 0.5%–5%) and imputed dosages were 0.7–0.9 in the European ancestry. Furthermore, mutational loads of rare alleles within genes obtained from imputation can confer high power [27]. In this study, we aimed to find risk genes for AD using gene-based analysis of rare alleles deduced from 1000 genomes and publicly available GWAS data.

## Materials and Methods

### Subjects

We used publicly available GWAS data from the Alzheimer’s Disease Neuroimaging Initiative (ADNI), Genetic Alzheimer’s Disease Associations (GenADA) study, Electronic Medical Records and Genomics (eMERGE), the National Institute on Aging Late Onset Alzheimer’s Disease (NIA-LOAD) family study, and the Framingham study. ADNI data were obtained from https://ida.loni.ucla.edu. GenADA (dbGaP accession number: phs000219.v1) [28], [29], eMERGE (dbGaP accession number: phs000234.v1), NIA-LOAD (dbGaP accession number: phs000168.v1), and the Framingham study (dbGaP accession number: phs000007.v16) data were downloaded from dbGaP (http://www.ncbi.nlm.nih.gov/gap). Subjects with European ancestry were included. After genotypic quality control (QC), missing phenotypic data exclusion, and ethnic group selection, 4171 cases and 9358 control were included in this study. Summaries about the studies are shown in Table 1. Additional information for each study were detained in File S1. The institutional review board of Ilsan hospital approved our study. Written informed consent was given by participants. In addition patient records were anonymized prior to analysis.

**Table 1:** Characteristics of studies.

Study	Genotyping platform	Case/Controlwith genetic data	Case/control after QC*	No of SNPs after QC	No of imputed SNPs§	No of imputed rare (MAF≤3%) SNPs§
**ADNI**	Illumina Human610-Quad	350/169	350/169	533479	16242208	8608819
**ADNI2**	Illumina GenomeStudio v2009.1	53/125	53/125	634701	14860121	7257490
**GenADA**	Affimetrix Mapping250K_NspMapping250K_Sty	782/806	779/803	432763	14441395	6863818
**eMERGE**	Illumina Human660W-Quad_v1_A	676/1843	632/1843	535401	16190257	8572925
**NIA-LOAD**	Illumina Human610-Quad_v1_B	2244/2320	2098/2095	542080	19568275	11943583
**Framingham**	Affimetrix Mapping250K_NspMapping250K_Sty	314/4711	259/4323	371114	16510848	8908801

* In addition to genotyping QC, we selected only European ancestry without missing information on age and sex.

§ SNPs with INFO≥0.4.

### Genotypic QC and imputation

We excluded alleles with low (<1%) MAF, low (<95%) call rate, and deviation of Hardy-Weinberg Equilibrium (*P*<10^–6^). The subjects with low (<95%) call rates, too high autosomal heterozygosity (false discovery rate, FDR<1%) and too high relatedness (identical-by-state, IBS>0.95) were excluded. For genotypic QC, we used the GenABEL package, v 1.69 [30].

After estimating haplotypes using SHAPEIT, v 1.0 [31], imputation with multi-population reference panels of 1000 genomes (phase I, release Mar 2012) was executed using IMPUTE2, v 2.2 with default parameters [32], [33]. We discarded imputated SNPs with INFO<0.4. The dosage data of imputation were used for further analyses. The dosage means the expected genotype score [34].

### Statistical analyses

In the association study, we adjusted for age, sex, years of education, and significant principle components (PCs) of the genetic stratification (File S1). For consistency across studies, years of education were categorized as follows: **1**, ≤ 4; **2**, 4< and ≤10; **3**, 11< and ≤ 15; **4**, >15 years according to the established methods of stratifications in the GenADA study. We imputed missing years of education to a mean value. The years of education was regarded as a continuous variable.

We performed a weighted, *Z* score based, fixed-effects, meta-analysis using METAL [35]. The effect sample size (*N_E_*) for meta-analysis is given in terms of numbers of AD (*N_AD_*) and of controls (*N_C_*), as follows [35]:
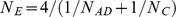



The forest plot was drawn using ‘rmeta’ R package.

*APOE* is the strongest risk gene among the known risk genes for AD. In several genome-wide association studies for AD [3], the top ranked genes could show false associations with AD, because they are within same LD block of *APOE* ε4. In addition, the pathogenesis of AD patients might be different between carriers and noncarriers of *APOE* ε4 [36]. Therefore, we examined the dependency on *APOE* ε4 genotype status by two ways. First, the results were compared after adjustment for *APOE* ε4 genotype status – the number of *APOE* ε4 allele in each individual. eMERGE and the Framingham study did not include data on *APOE* ε4 genotype status. Therefore, we used imputed dosages of *APOE* ε4 for these two studies (Table S1 in File S1). Second, the collinearity between selected genes and *APOE* ε4 genotype status was examined.

### Gene-based rare allele analysis

In this study, gene-based rare allele analysis means accumulations of rare alleles within the same coding region implemented in GRANVIL [27]. The definition of gene boundaries was based on the UCSC genome browser (build 37). The Framingham study showed inflated type I error and skewed results (Figures S1 and S2 in File S1). Therefore, we need to adjust for genetic stratification of the Framingham study using another algorithm implemented in GenABEL v1.69 and ProbABEL v0.30 [30], [37] (Figure S2 in File S1). For gene-based analysis of the Framingham study, we need to make computer program for ourselves. We made a dosage of a gene (*D*) similar to an allele’s dosage in the Framingham study, as follows [27].
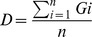



Where *Gi* is a dosage of the *i*th SNP and *n* is a number of rare alleles within a gene that were used in the analysis.

**Figure 1:**
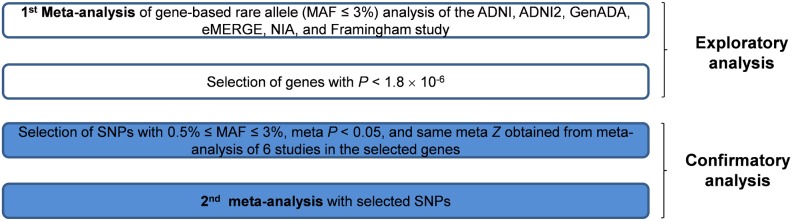
The overall scheme of this study.

Analyses proceeded in two steps. The overall study scheme is shown in Figure 1. We performed the first meta-analysis to select genes with genome-wide significance. The genome-wide significance was defined as significance of *P*<1.8×10^−6^ (0.05/number of genes in human genome in UCSC genome browser (build 37) = 0.05/28517). However, there are three shortcomings in the gene-based rare allele analysis using imputation. First, it is difficult to interpret if there are a lot of rare alleles in a gene. Second, by pooling risk and protective alleles, power can be decreased. However, considering such directions before selecting candidate genes, overinflation of type I error can be problematic. Third the accuracy of imputation can be decreased in rare alleles with very low MAF. We performed confirmatory analysis (the second meta-analysis) with selected SNPs We did confirmatory analysis, according to two reasons. First, if we could test genetic risk factors with a small number of SNPs, it would be more convenient for genotyping and interpretation. Therefore, we selected several risk SNPs in the finally selected gene according to meta *P* and meta *Z* (*P*<0.05 and Z>0) after performing classical SNP based GWAS and meta-analysis. Second, we excluded rare variants with MAF<0.5%, because the imputation accuracy decreases in very low MAF [20].

## Results

### The first meta-analysis

**Figure 2:**
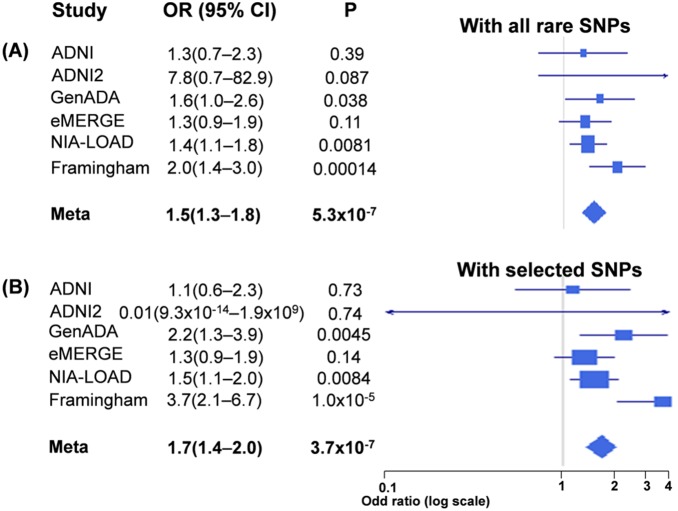
Forest plots showing the association of *ZNF628* with AD. Results are (A) with all rare SNPs (the first meta-analysis) and (B) with only selected risk SNPs (the second meta-analysis). The weight of each study was calculated by 4/(1/*N_AD_*+1/*N_C_*), where *N_AD_* and *N_C_* are numbers of AD and controls, respectively [35].

In the meta-analysis, *ZNF628* had genome-wide significance (meta *P* = 5.3×10^–7^ [OR 1.5, 95% CI 1.3–1.8]) (Table 2 and Figure 2A). SNPs in *ZNF628* used in this study are summarized in Table S2 in File S1. In addition, *APOE* had also genome-wide significance (meta *P* = 1.4×10^–6^). Other genes with high significances, but not with genome-wide significance were *TOMM40, MMP1, NAPRT1, TREM2,* and *CBLB*.

**Table 2:** The highly ranked seven genes in the first meta-analyses.

Gene	CHR	Start	not adjusted for *APOE* ε4			adjusted for *APOE* ε4	
			Meta *Z* *	Meta *P*	direction§	Meta *Z*	Meta *P*
***ZNF628***	19	55987698	5.0	5.3×10^–7^	++++++	5.2	1.3×10^–7^
***APOE***	19	45409038	4.8	1.4×10^–6^	+?+++–	2.3	0.023
***TOMM40***	19	45394477	4.1	4.0×10^–5^	+–++++	2.3	0.018
***MMP1***	11	102654407	4.0	6.6×10^–5^	–++++	4.1	4.0×10^–5^
***NAPRT1***	8	144656956	3.9	7.8×10^–5^	++++++	4.5	8.0×10^–6^
***TREM2***	6	41126245	3.8	1.2×10^–4^	++++++	4.6	3.7×10^–6^
***CBLB***	3	105438891	3.8	1.5×10^–4^	++++++	3.2	0.0015

We show the highly ranked genes (meta *P*<2.0×10^–4^) in the first meta-analysis in this table.

Protein names: zinc finger protein 628, ZNF628; apolipoprotein E, APOE; translocase of outer mitochondrial membrane 40, TOMM40; matrix metallopeptidase 1, MMP1; nicotinate phosphoribosyltransferase domain containing 1, NAPRT1; triggering receptor expressed on myeloid cells 2, TREM2; Cbl proto-oncogene B, E3 ubiquitin protein ligase, CBLB.

* Larger absolute *Z* score represents smaller *P* and the direction of the *Z* score represents the direction of risk [35].

§ The signs mean those of the *Z* score of each study. The question mark represents missing data in the study because of low INFO or high MAF. The order of the signs is ADNI, ADNI2, GenADA, eMERGE, NIA-LOAD, and Framingham study.

### Dependency on *APOE* ε4 genotype status

We examined the dependencies of the selected genes by adjusting for *APOE* ε4 (Table 2). The significance of *ZNF628* was remained, even after adjustment. However, the significance of *APOE* decreased after adjustment for *APOE* ε4 (after adjustment, *P* value of *APOE* increased to 0.023).

Additionally, the collinearity between *ZNF628* and *APOE* ε4 genotype status were examined based on the variance inflation factor (VIF, Table S3 in File S1). The VIFs of all studies were approximately 1.

### Meta-analysis with selected risk SNPs (the confirmatory second analysis)

**Figure 3:**
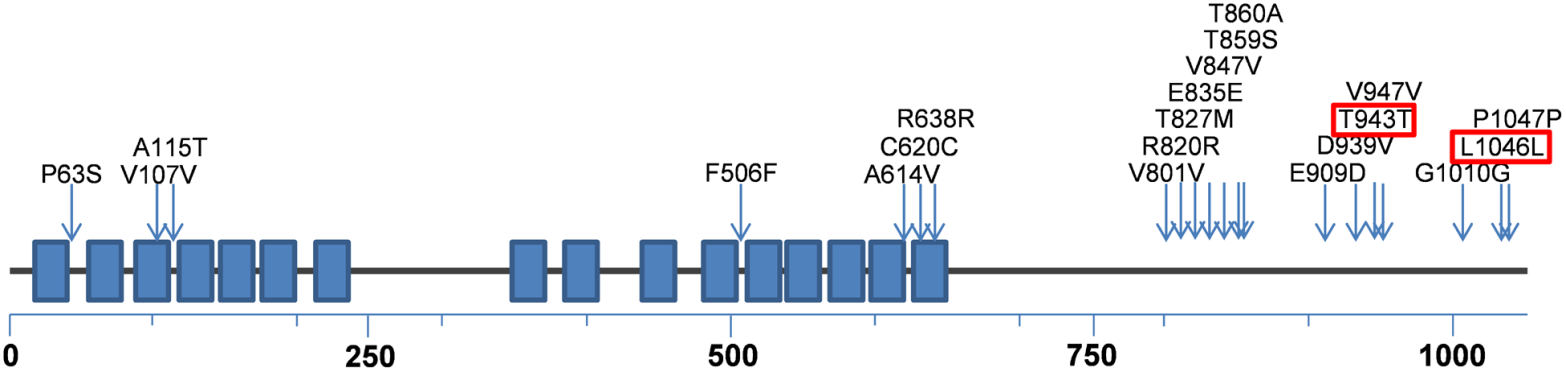
Schematic representation of *ZNF628* with locations of SNPs used in gene-based rare allele analysis in this study. *ZNF628* is a protein 1059 amino acids long. We briefly showed the domains (boxes) and the locations of SNPs (arrows) in a schematic linear structure of *ZNF628*. Blue boxes denote C2H2-type zinc finger domains. dbSNP ID can be found in Table S2 in File S1. SNPs within red boxes were used in the second analysis.

For a more applicable clinical approach, we identified significant risk SNPs by meta *P* and meta *Z* scores. Furthermore, considering the imputation accuracy [20], we selected SNPs with 0.5% ≤ MAF≤3%. Two risk SNPs (dbSNP ID: rs112407198 and rs73057174) selected within *ZNF628* were synonymous SNPs (Figure 3). As shown in Figure 2B and Table 3, gene-based rare allele analysis using only selected SNPs had genome-wide significance with moderately high effect size (meta *P* = 3.7×10^–7^ [OR 1.7, 95% CI 1.4–2.0]).

**Table 3:** Results of the second meta-analysis (confirmatory analysis).

	rs112407198 (position: 19∶55995401)						rs73057174 (position: 19∶55995710)						Gene-based	
Study	MAF	INFO	not adjusted for *APOE* ε4		adjusted for *APOE* ε4		MAF	INFO	not adjustedfor *APOE* ε4		adjusted for*APOE* ε4		*P* not adjustedfor *APOE* ε4	*P* adjustedfor *APOE* ε4
			beta	*P*	beta	*P*			beta	*P*	beta	*P*		
**ADNI**	0.011	0.78	0.29	0.70	0.09	0.91	0.029	0.91	0.08	0.85	0.39	0.40	0.73	0.43
**ADNI2**	0.009	0.82	–8.7	0.79	0.50	0.87	0.031	0.95	–0.30	0.63	–0.083	0.90	0.74	0.55
**GenADA**	0.011	0.47	1.99	0.0052	1.93	0.0079	0.022	0.67	0.52	0.11	0.59	0.093	0.0045	0.0049
**eMERGE**	0.012	0.72	0.16	0.65	0.11	0.75	0.022	0.90	0.32	0.15	0.26	0.26	0.14	0.26
**NIA**	0.013	0.74	0.24	0.39	0.079	0.80	0.024	0.90	0.46	0.011	0.62	0.0015	0.0084	0.0042
**Framingham**	0.011	0.41	1.31	0.0079	1.34	0.0066	0.019	0.55	1.35	0.00026	1.34	0.00029	1.0×10^–5^	9.7×10^–6^
**Meta**				0.0043		0.019				2.8×10^–5^		2.7×10^–6^	3.7×10^–7^	2.8×10^–7^

Key: MAF, minor allele frequency; INFO, imputation quality score made by IMPUTE2.

### Gene-based rare allele analyses for the genes known to be associated with AD

Interestingly, rare alleles in *APOE* and *TREM2* showed significantly high association with AD (Table 2). Thus, we tested rare alleles of other known genes associated with AD. The most highly ranked nine genes in the AlzGene database [38] (*ABCA7, PICALM, CLU, MS4A6A/MS4AE, CD33, BIN1, CR1, and CD2AP*) were selected for the test. Based on the meta-analysis, only *BIN1* had significance (meta *P* = 0.046), but did not reach to genome-wide significance level (Table 4).

**Table 4:** Results of gene-based rare allele analysis top ranking genes in the AlzGene database.

Gene	CHR	start	Meta *Z*	Meta *P*	Directions*
***ABCA7***	19	1040101	0.2	0.8539	+++–+
***PICALM***	11	85668485	–0.4	0.7068	–?+–+–
***CLU***	8	27454450	–1.1	0.2554	–++–
***MS4A6A***	11	59939080	0.5	0.6377	–+–+–+
***CD33***	19	51728334	–0.4	0.7185	–++–
***BIN1***	2	127805606	2.0	0.0460	++++–
***MS4A4E***	11	59980567	0.5	0.6377	–+–+–+
***CR1***	1	207669472	0.7	0.5050	++–+–+
***CD2AP***	6	47445524	–1.1	0.2593	–+–+

Protein names: ATP-binding cassette, sub-family A, ABCA7; phosphatidylinositol binding clathrin assembly protein, PICALM; clusterin, CLU; membrane-spanning 4-domains, subfamily A, member 6A, MS4A6A; CD33 molecule, CD33; bridging integrator 1, BIN1; putative membrane-spanning 4-domains subfamily A member 4E, MS4A4E; complement component (3b/4b) receptor 1, CR1; CD2-associated protein, CD2AP.

* The signs represent those of the *Z* score of each study. The question mark represents missing data in the study because of low INFO or high MAF. The order of the signs is ADNI, ADNI2, GenADA, eMERGE, NIA, and Framingham study.

## Discussion

We performed meta-analysis with publicly available genetic studies of AD with imputed rare (MAF≤3%) alleles. *ZNF628* was identified to have significant association with AD. Additionally, our rare allele analysis revealed the significant association of *APOE* and *TREM2* with AD, which suggested that our results were valid and that these genes require further study [39], [40].

*ZNF628* is a C2H2-zinc finger protein, a type of transcription factors [41] consisting of three exons. C2H2-type zinc finger proteins are known to be essential for normal growth and development [41]. *ZNF628* is found in mammals, but not Zebra fish or C. elegans [41]. *ZNF628* is evenly expressed in various tissues including brain [42], [43]. *ZNF628* is conserved among mammals and seems to be functionally important [41]. The possible DNA binding site is the sequence motif – C/GA/TA/TGGTTGGTTGC [41]. As this time, the target proteins and related human disorders associated with *ZNF628* have not been reported. It is possible that the rare alleles in *ZNF628* change the expression levels of certain proteins related to AD pathogenesis.

In the selected allele analysis of *ZNF628* (the second confirmatory analysis), *P* and *Z* values of two SNPs (rs112407198 and rs73057174) reached the criteria of *P*<0.05 and *Z*>0. These SNPs are located outside the C2H2-type zinc finger domains and synonymous SNPs (Figure 3). The synonymous mutations are known to change the protein expression level and conformation [44] by affecting mRNA structure [45] or changing the time of cotranslational folding [46]. The altered expression levels or structure of *ZNF628* could affect the expression level of other proteins.

There were no dependencies between *ZNF628* and *APOE* ε4 genotype status. *ZNF628* is separated from *APOE* by more than 10^8 ^bp, although they are both located on chromosome 19. Therefore, *ZNF628* is not included in same LD block with *APOE* ε4. *ZNF628* did not lose its significance in meta-analysis even after adjustment for *APOE* ε4 genotype status. Therefore, *ZNF628* appears to be related with AD independently from *APOE* ε4. In contrast, the significance of *APOE* was affected by *APOE* ε4. The association of the rare alleles in *APOE* with AD was highly significant (*P* = 1.4×10^–6^) with AD, although this significance disappeared after adjusting for *APOE* ε4. This suggested that rare alleles in the same LD block with *APOE* ε4 conferred significant association with AD.

Other risk genes that have been found in GWAS targeting common alleles were not replicated in our gene-based rare allele analysis. Only *TREM2*, which has been identified in previous studies targeting rare alleles, showed high significance levels [39], [40]. Common alleles with small effect sizes have been explained by synthetic association of rare alleles [47], [48]. Recently, however, this hypothesis was not confirmed in a large-scale study of seven common immune diseases [49]. Similarly, we could not show association of rare alleles within the known genes with AD.

There are several limitations in this study. First, a replication study with real genotyping is required. However, 1000 genomes-based imputations can enable us to find refined and novel signals [50]. Furthermore, the sample size and power can be increased by imputation [51] and meta-analysis [52]. Our gene-based rare variant analysis by imputation have comparable high power with re-sequencing analysis, especially with a large number of sample size [27]. Second, rare alleles analysis of *ZNF628* of this study was performed in White populations. Although this result should be replicated in different populations, it is difficult to identify. The two important selected SNPs of our study, rs11247198 and rs73057174, have not been reported in Asian populations, whereas higher MAF has been identified in Black populations (especially in the Bushmen). Third, current methods of rare allele analysis still have problems and need more powerful and consistent methods [53]. The simulated studies using 20 different tools did not generate consistent results [54]. Therefore, simulation studies to identify methods that generate the optimal results are required [53]. Additionally, the directions of SNPs for related diseases are not usually considered [53]. Lastly, the SNPs in introns could not be considered because of limited our computational resources.

In conclusion, we observed a noble association between *ZNF628* and AD. Considering the biological role of the *ZNF628* protein, it may contribute to AD by regulating various AD-related proteins expressions. Functional studies to elucidate its contribution to AD pathogenesis are required. Additionally, further studies addressing different populations should be replicated to assess the value of the *ZNF628* rare allele as a genetic biomarker of AD.

## Supporting Information

File S1 **Supplement text, tables, and figures.**
(DOCX)

## References

[1] Pedersen NL, Posner SF, Gatz M (2001) Multiple-threshold models for genetic influences on age of onset for Alzheimer disease: findings in Swedish twins. Am J Med Genet 105: 724–728. 11803520 10.1002/ajmg.1608

[2] Gatz M, Reynolds CA, Fratiglioni L, Johansson B, Mortimer JA, et al (2006) Role of genes and environments for explaining Alzheimer disease. Arch Gen Psychiatry 63: 168–174. 16461860 10.1001/archpsyc.63.2.168

[3] Harold D, Abraham R, Hollingworth P, Sims R, Gerrish A, et al (2009) Genome-wide association study identifies variants at CLU and PICALM associated with Alzheimer’s disease. Nat Genet 41: 1088–1093. 19734902 10.1038/ng.440 PMC2845877

[4] Lambert JC, Heath S, Even G, Campion D, Sleegers K, et al (2009) Genome-wide association study identifies variants at CLU and CR1 associated with Alzheimer’s disease. Nat Genet 41: 1094–1099. 19734903 10.1038/ng.439

[5] Hollingworth P, Harold D, Sims R, Gerrish A, Lambert JC, et al (2011) Common variants at ABCA7, MS4A6A/MS4A4E, EPHA1, CD33 and CD2AP are associated with Alzheimer’s disease. Nat Genet 43: 429–435. 21460840 10.1038/ng.803 PMC3084173

[6] Naj AC, Jun G, Beecham GW, Wang LS, Vardarajan BN, et al (2011) Common variants at MS4A4/MS4A6E, CD2AP, CD33 and EPHA1 are associated with late-onset Alzheimer’s disease. Nat Genet 43: 436–441. 21460841 10.1038/ng.801 PMC3090745

[7] Seshadri S, Fitzpatrick AL, Ikram MA, DeStefano AL, Gudnason V, et al (2010) Genome-wide analysis of genetic loci associated with Alzheimer disease. JAMA 303: 1832–1840. 20460622 10.1001/jama.2010.574 PMC2989531

[8] Bertram L (2011) Alzheimer’s genetics in the GWAS era: a continuing story of 'replications and refutations’. Curr Neurol Neurosci Rep 11: 246–253. 21487954 10.1007/s11910-011-0193-z

[9] Sullivan PF, Daly MJ, O’Donovan M (2012) Genetic architectures of psychiatric disorders: the emerging picture and its implications. Nat Rev Genet 13: 537–551. 22777127 10.1038/nrg3240 PMC4110909

[10] Reich DE, Lander ES (2001) On the allelic spectrum of human disease. Trends Genet 17: 502–510. 11525833 10.1016/s0168-9525(01)02410-6

[11] Schork NJ, Murray SS, Frazer KA, Topol EJ (2009) Common vs. rare allele hypotheses for complex diseases. Curr Opin Genet Dev 19: 212–219. 19481926 10.1016/j.gde.2009.04.010 PMC2914559

[12] Manolio TA, Collins FS, Cox NJ, Goldstein DB, Hindorff LA, et al (2009) Finding the missing heritability of complex diseases. Nature 461: 747–753. 19812666 10.1038/nature08494 PMC2831613

[13] Asimit J, Zeggini E (2010) Rare variant association analysis methods for complex traits. Annu Rev Genet 44: 293–308. 21047260 10.1146/annurev-genet-102209-163421

[14] van de Ven JP, Nilsson SC, Tan PL, Buitendijk GH, Ristau T, et al (2013) A functional variant in the CFI gene confers a high risk of age-related macular degeneration. Nat Genet 45: 813–817. 23685748 10.1038/ng.2640

[15] Wheeler E, Huang N, Bochukova EG, Keogh JM, Lindsay S, et al (2013) Genome-wide SNP and CNV analysis identifies common and low-frequency variants associated with severe early-onset obesity. Nat Genet 45: 513–517. 23563609 10.1038/ng.2607 PMC4106235

[16] Huyghe JR, Jackson AU, Fogarty MP, Buchkovich ML, Stancakova A, et al (2013) Exome array analysis identifies new loci and low-frequency variants influencing insulin processing and secretion. Nat Genet 45: 197–201. 23263489 10.1038/ng.2507 PMC3727235

[17] Styrkarsdottir U, Thorleifsson G, Sulem P, Gudbjartsson DF, Sigurdsson A, et al (2013) Nonsense mutation in the LGR4 gene is associated with several human diseases and other traits. Nature 497: 517–520. 23644456 10.1038/nature12124

[18] Bodmer W, Bonilla C (2008) Common and rare variants in multifactorial susceptibility to common diseases. Nat Genet 40: 695–701. 18509313 10.1038/ng.f.136 PMC2527050

[19] Tennessen JA, Bigham AW, O’Connor TD, Fu W, Kenny EE, et al (2012) Evolution and functional impact of rare coding variation from deep sequencing of human exomes. Science 337: 64–69. 22604720 10.1126/science.1219240 PMC3708544

[20] Abecasis GR, Auton A, Brooks LD, DePristo MA, Durbin RM, et al (2012) An integrated map of genetic variation from 1,092 human genomes. Nature 491: 56–65. 23128226 10.1038/nature11632 PMC3498066

[21] MacArthur DG, Balasubramanian S, Frankish A, Huang N, Morris J, et al (2012) A systematic survey of loss-of-function variants in human protein-coding genes. Science 335: 823–828. 22344438 10.1126/science.1215040 PMC3299548

[22] Rovelet-Lecrux A, Legallic S, Wallon D, Flaman JM, Martinaud O, et al (2012) A genome-wide study reveals rare CNVs exclusive to extreme phenotypes of Alzheimer disease. Eur J Hum Genet 20: 613–617. 22166940 10.1038/ejhg.2011.225 PMC3355247

[23] Jonsson T, Atwal JK, Steinberg S, Snaedal J, Jonsson PV, et al (2012) A mutation in APP protects against Alzheimer’s disease and age-related cognitive decline. Nature 488: 96–99. 22801501 10.1038/nature11283

[24] Cruchaga C, Karch CM, Jin SC, Benitez BA, Cai Y, et al (2014) Rare coding variants in the phospholipase D3 gene confer risk for Alzheimer’s disease. Nature 505: 550–554. 24336208 10.1038/nature12825 PMC4050701

[25] Walsh T, McClellan JM, McCarthy SE, Addington AM, Pierce SB, et al (2008) Rare structural variants disrupt multiple genes in neurodevelopmental pathways in schizophrenia. Science 320: 539–543. 18369103 10.1126/science.1155174

[26] Shea J, Agarwala V, Philippakis AA, Maguire J, Banks E, et al (2011) Comparing strategies to fine-map the association of common SNPs at chromosome 9p21 with type 2 diabetes and myocardial infarction. Nat Genet 43: 801–805. 21775993 10.1038/ng.871 PMC4096898

[27] Magi R, Asimit JL, Day-Williams AG, Zeggini E, Morris AP (2012) Genome-Wide Association Analysis of Imputed Rare Variants: Application to Seven Common Complex Diseases. Genet Epidemiol 36: 785–796. 22951892 10.1002/gepi.21675 PMC3569874

[28] Filippini N, Rao A, Wetten S, Gibson RA, Borrie M, et al (2009) Anatomically-distinct genetic associations of APOE epsilon4 allele load with regional cortical atrophy in Alzheimer’s disease. Neuroimage 44: 724–728. 19013250 10.1016/j.neuroimage.2008.10.003

[29] Li H, Wetten S, Li L, St Jean PL, Upmanyu R, et al (2008) Candidate single-nucleotide polymorphisms from a genomewide association study of Alzheimer disease. Arch Neurol 65: 45–53. 17998437 10.1001/archneurol.2007.3

[30] Aulchenko YS, Ripke S, Isaacs A, van Duijn CM (2007) GenABEL: an R library for genome-wide association analysis. Bioinformatics 23: 1294–1296. 17384015 10.1093/bioinformatics/btm108

[31] Delaneau O, Marchini J, Zagury JF (2012) A linear complexity phasing method for thousands of genomes. Nat Methods 9: 179–181. 10.1038/nmeth.1785 22138821

[32] Howie B, Fuchsberger C, Stephens M, Marchini J, Abecasis GR (2012) Fast and accurate genotype imputation in genome-wide association studies through pre-phasing. Nat Genet 44: 955–959. 22820512 10.1038/ng.2354 PMC3696580

[33] Howie B, Marchini J, Stephens M (2011) Genotype imputation with thousands of genomes. G3 (Bethesda) 1: 457–470. 22384356 10.1534/g3.111.001198 PMC3276165

[34] Zheng J, Li Y, Abecasis GR, Scheet P (2011) A comparison of approaches to account for uncertainty in analysis of imputed genotypes. Genet Epidemiol 35: 102–110. 21254217 10.1002/gepi.20552 PMC3143715

[35] Willer CJ, Li Y, Abecasis GR (2010) METAL: fast and efficient meta-analysis of genomewide association scans. Bioinformatics 26: 2190–2191. 20616382 10.1093/bioinformatics/btq340 PMC2922887

[36] Rhinn H, Fujita R, Qiang L, Cheng R, Lee JH, et al (2013) Integrative genomics identifies APOE epsilon4 effectors in Alzheimer’s disease. Nature 500: 45–50. 23883936 10.1038/nature12415

[37] Aulchenko YS, Struchalin MV, van Duijn CM (2010) ProbABEL package for genome-wide association analysis of imputed data. BMC Bioinformatics 11: 134. 20233392 10.1186/1471-2105-11-134 PMC2846909

[38] Bertram L, McQueen MB, Mullin K, Blacker D, Tanzi RE (2007) Systematic meta-analyses of Alzheimer disease genetic association studies: the AlzGene database. Nat Genet 39: 17–23. 17192785 10.1038/ng1934

[39] Jonsson T, Stefansson H, Steinberg S, Jonsdottir I, Jonsson PV, et al (2013) Variant of TREM2 associated with the risk of Alzheimer’s disease. N Engl J Med 368: 107–116. 23150908 10.1056/NEJMoa1211103 PMC3677583

[40] Guerreiro R, Wojtas A, Bras J, Carrasquillo M, Rogaeva E, et al (2013) TREM2 variants in Alzheimer’s disease. N Engl J Med 368: 117–127. 23150934 10.1056/NEJMoa1211851 PMC3631573

[41] Chen GY, Muramatsu H, Ichihara-Tanaka K, Muramatsu T (2004) ZEC, a zinc finger protein with novel binding specificity and transcription regulatory activity. Gene 340: 71–81. 15556296 10.1016/j.gene.2004.06.016

[42] Wu C, Orozco C, Boyer J, Leglise M, Goodale J, et al (2009) BioGPS: an extensible and customizable portal for querying and organizing gene annotation resources. Genome Biol 10: R130. 19919682 10.1186/gb-2009-10-11-r130 PMC3091323

[43] Derrien T, Johnson R, Bussotti G, Tanzer A, Djebali S, et al (2012) The GENCODE v7 catalog of human long noncoding RNAs: analysis of their gene structure, evolution, and expression. Genome Res 22: 1775–1789. 22955988 10.1101/gr.132159.111 PMC3431493

[44] Sauna ZE, Kimchi-Sarfaty C (2011) Understanding the contribution of synonymous mutations to human disease. Nat Rev Genet 12: 683–691. 21878961 10.1038/nrg3051

[45] Nackley AG, Shabalina SA, Tchivileva IE, Satterfield K, Korchynskyi O, et al (2006) Human catechol-O-methyltransferase haplotypes modulate protein expression by altering mRNA secondary structure. Science 314: 1930–1933. 17185601 10.1126/science.1131262

[46] Kimchi-Sarfaty C, Oh JM, Kim IW, Sauna ZE, Calcagno AM, et al (2007) A “silent” polymorphism in the MDR1 gene changes substrate specificity. Science 315: 525–528. 17185560 10.1126/science.1135308

[47] Dickson SP, Wang K, Krantz I, Hakonarson H, Goldstein DB (2010) Rare variants create synthetic genome-wide associations. PLoS Biol 8: e1000294. 20126254 10.1371/journal.pbio.1000294 PMC2811148

[48] Cirulli ET, Goldstein DB (2010) Uncovering the roles of rare variants in common disease through whole-genome sequencing. Nat Rev Genet 11: 415–425. 20479773 10.1038/nrg2779

[49] Hunt KA, Mistry V, Bockett NA, Ahmad T, Ban M, et al (2013) Negligible impact of rare autoimmune-locus coding-region variants on missing heritability. Nature 498: 232–235. 23698362 10.1038/nature12170 PMC3736321

[50] Huang J, Ellinghaus D, Franke A, Howie B, Li Y (2012) 1000 Genomes-based imputation identifies novel and refined associations for the Wellcome Trust Case Control Consortium phase 1 Data. Eur J Hum Genet 20: 801–805. 22293688 10.1038/ejhg.2012.3 PMC3376268

[51] de Bakker PI, Ferreira MA, Jia X, Neale BM, Raychaudhuri S, et al (2008) Practical aspects of imputation-driven meta-analysis of genome-wide association studies. Hum Mol Genet 17: R122–128. 18852200 10.1093/hmg/ddn288 PMC2782358

[52] Skol AD, Scott LJ, Abecasis GR, Boehnke M (2007) Optimal designs for two-stage genome-wide association studies. Genet Epidemiol 31: 776–788. 17549752 10.1002/gepi.20240

[53] Bansal V, Libiger O, Torkamani A, Schork NJ (2010) Statistical analysis strategies for association studies involving rare variants. Nat Rev Genet 11: 773–785. 20940738 10.1038/nrg2867 PMC3743540

[54] Bansal V, Libiger O, Torkamani A, Schork NJ (2011) An application and empirical comparison of statistical analysis methods for associating rare variants to a complex phenotype. Pac Symp Biocomput: 76–87. 10.1142/9789814335058_0009 PMC5017238 21121035

