# Supplementary material for: Gene-Based Rare Allele Analysis Identified a Risk Gene of Alzheimer’s Disease
Source: PLoS One. 2014 Oct 20;9(10):e107983. doi: 10.1371/journal.pone.0107983 (PMC4203677; doi:10.1371/journal.pone.0107983)
Supplement: File S1 — Supplement text, tables, and figures. (DOCX) [file pone.0107983.s001.docx]

**Participants**

*Alzheimer’s Disease Neuroimaging Initiatives (ADNI)*

ADNI is a multicenter longitudinal study. We refer to diagnoses in November 2012. The definition of normal cognition (NC) was given as follows: no memory complaints and normal objective memory performance, Mini Mental State Exam (MMSE) scores of 24–30, and a clinical dementia rating (CDR) scale score of 0. Patients with AD sufficiently met the probable criteria of AD from the National Institute of Neurological and Communicative Disorders and Stroke and the Alzheimer’s Disease and Related Disorders Association (NINCDS-ADRDA) [[1](#_ENREF_1)]. Because ADNI included early stage AD, patients of AD had MMSE scores of 20–26 and a sum-of-box CDR score of 1–9. ADNI has three phases : ADNI1, ADNI GO (Grand Opportunity), and ADNI2. ADNI GO included only patients with mild cognitive impairment, and we did not include ADNI GO in this study. We separately analyzed the two phases of ADNI because DNA chip platforms differed between ADNI and ADNI2. Patients with serious neurological diseases other than AD, brain lesions, or head trauma were excluded. Patients taking psychoactive medications were also excluded. ADNI subjects included 350 cases and 169 controls. ADNI2 included 53 cases and 125 controls.

*Genetic Alzheimer’s Disease Associations (GenADA) study*

GenADA is a multicenter case-control study conducted by GalxoSmithKline Inc. and nine medical centers in Canada. AD patients met the NINCDS-ADRDA criteria for probable AD. Patients with current psychiatric diseases were excluded. The ethnicity-, gender-, and age-matched controls have following criteria: (1) no memory problems, (2) higher MMSE scores than cut-off values for dementia considering age and education level, (3) Dementia Rating Scale (DRS) – 2 (Age and Education adjusted MOANS scale score (AEMSS) of 9 or higher (after adjustment for age and education); (4) clock test (11:10) ≥ 14, and (5) no impairment in the seven instrumental ADL questions from the Duke Older American Resources and Services Procedures caused by cognitive decline. Cases also did not have current psychiatric diseases. In total, there were 782 cases and 806 controls in GenADA study.

*Electronic Medical Records and Genomics (eMERGE) study*

eMERGE is a case-control stud with four population-based sources : the University of Washington Alzheimer Disease Patient Registry (ADPR), Adult Changes in Thought (ACT) study, Marshfield Clinic Personalized Medicine Research Project (PMRP), and Vanderbilt University’s DNA databank (BioVU). The genetic studies of AD included ADPR and ACT cases and ACT controls. AD was diagnosed according to NINCDS-ADRDA criteria. Controls in the ACT study were evaluated using the Cognitive Abilities Screening Instrument (CASI) every two years. The ages of controls were ≥ 65 years old. Diagnosis of the controls was stratified according to CASI scores as follows: – (1) a baseline CASI > 86, (2) a baseline CASI 80–86, two months follow-up CASI > 86, and normal Informant Questionnaire upon Cognitive Decline in the Elderly (IQCODE) score, or (3) a baseline CASI < 80 and no dementia on complete diagnostic evaluation. Exclusion criteria for controls were residents of nursing homes, participants in other studies, and subjects with diagnosis of dementia. Subjects who did not agree to submit their information to dbGaP were also excluded. In total, 676 cases and 1843 controls were included in the eMERGE study.

*National Institute on Aging Late Onset Alzheimer’s Disease (NIA-LOAD) Family study*

NIA-LOAD is a family, longitudinal, case-control study. Cases were selected based on autopsy [[2-5](#_ENREF_2)] or probable AD according to NINCDS-ADRDA criteria [[1](#_ENREF_1)]. Only cases with age at onset or age at diagnosis of ≥ 60 years were selected. The controls were also ≥ 60 years old. Controls were included only if they had no subjective memory complaints or cognitive decline. A neuropsychological test, semiquantitative neurological or psychiatric examination was evaluated in selecting controls. Additionally, the controls did not have other past/current neurological or psychiatric diseases such as schizophrenia, biopolar disorder, major depression, obsessive-compulsive disorder, anxiety/panic disorder, Parkinson disease, Alzheimer’s disease, multiple sclerosis, brain tumor, and Huntington’s disease. They had no history of life-threatening conditions. The numbers of cases and controls were 2244 and 2320, respectively.

*Framingham study*

The Framingham study is a prospective, community-based family study. Cases were selected according to probable or possible AD based on NINCDS-ADRDA criteria [[1](#_ENREF_1)]. There were 314 cases and 4711 controls.

**Adjusting for the genetic stratification**

The principal component (PC) analysis for the genetic stratification was performed using GenABEL, v 1.69 [[6](#_ENREF_6)]. Inclusion of PCs not significantly associated with phenotypes can reduce the power [[7](#_ENREF_7)]. Therefore, we selected PCs that were significantly associated with phenotypes using multiple logistic regressions adjusting for age, sex, and years of education. The significant PCs according to studies are shown in Table S3. Q-Q plot of the Framingham study still had inflation even after adjustment for three leading PCs (Figures. S1 and S2). The Framingham study is a community-based family study, and such inflation could arise from inclusion of genetically related subjects. Therefore, we adjusted for genetic stratification of the Framingham study using the ‘mmscore’ function implemented in GenABEL v1.69 and ProbABEL v0.30 [[6](#_ENREF_6), [8](#_ENREF_8)].

**Quantile-quantile (Q-Q) plots**

Because *APOE*’s role as an AD risk gene has been consistently replicated across studies on AD genetics [[9](#_ENREF_9)], we could estimate the degrees of the quality control based on the results of SNPs located close to *APOE*. *APOE* is at position 45409038 of chromosome 19. In five studies (excluding the Framingham study), the SNPs most significantly associated with AD were located in close proximity to *APOE*, suggesting good quality control in these studies (Figure S1). In contrast, the Framingham study showed inflated type I error and skewed results (Figures. S1 and S2). In the GenADA study, the genotyping platform was the same as that of the Framingham study, and the most significant SNP associated with AD was rs41377151 located at position 45422946 of chromosome 19. After adjustment of the Framingham study using a method implemented in GenABEL, the problems of inflation of type I error and skewed results disappeared (Figure. S2) [[6](#_ENREF_6)]. The significance of rs41377151 in the Framingham study increased to the most significant level (before adjustment, *P* = 9.3 × 10^–6^; after adjustment, *P* = 2.4 × 10^–11^). Q-Q plots were drawn using Galaxy [[10](#_ENREF_10)].

**Figure S1. Q-Q plots.** The expected *P* values under the null distribution are denoted by red lines. The observed *P* values are plotted with black dots. The SNP with the best result in each study is indicated with grey letters denoting dbSNP ID and the location in chromosome.

**Figure S2. Q-Q plots of the Framingham study.** The results (A) before and (B) after adjustment for genetic stratification using the ‘mmscore’ function of GenABEL. The SNP with the best result of each study is indicated with grey letters denoting dbSNP ID and the location in chromosome.

**Table S1**. **Imputation qualities of *APOE* ɛ4 (rs429358) and ɛ2 (rs7412)**

| **Study** | **Parameters** | **rs429358** | **rs7412** |
| --- | --- | --- | --- |
| **eMERGE** | MAF | 15.30% | 8.00% |
|  | INFO | 0.936 | 0.912 |
| **Framingham** | MAF | 11.70% | 9.10% |
|  | INFO | 0.441 | 0.540 |

eMERGE and Framingham studies do not have genotyping information on *APOE* ɛ4 and ɛ2. We used the imputed *APOE* ɛ4 in the analysis of the studies.

Key: MAF, minor allele frequency; INFO, imputation quality score of IMPUTE2

**Table S2. Rare alleles of *ZNF628* used in this study.**

| **SNP name** | **Amino acid change & position** | **Position** | **ADNI (14)**^*^ | | **ADNI2 (6)** ^*^ | | **GenADA (11)** ^*^ | | **eMERGE (9)**^*^ | | **NIA (19)**^*^ | | **Framingham (10)**^*^ | | **Meta** | | |
| --- | --- | --- | --- | --- | --- | --- | --- | --- | --- | --- | --- | --- | --- | --- | --- | --- | --- |
|  |  |  | **MAF** | **INFO** | **MAF** | **INFO** | **MAF** | **INFO** | **MAF** | **INFO** | **MAF** | **INFO** | **MAF** | **INFO** | ***Z*** | ***P*** | **Direction**^§^ |
| rs200942784 | P63S | 55992759 | 0.003 | 0.66 | 0.0 | 0.045 | 0.002 | 0.41 | 0.001 | 0.52 | 0.001 | 0.48 | 0.002 | 0.42 | -0.7 | 0.48 | -+-+-- |
| rs147923255 | V109V | 55992899 | 0.0 | 0.071 | 0.0 | 0.029 | 0.002 | 0.33 | 0.0 | 0.18 | 0.002 | 0.79 | 0.004 | 0.47 | 2.1 | 0.034 | +-++++ |
| rs180681662 | A115T | 55992915 | 0.002 | 0.56 | 0.002 | 0.61 | 0.001 | 0.64 | 0.001 | 0.54 | 0.001 | 0.64 | 0.001 | 0.46 | -0.4 | 0.68 | +--+-- |
| rs202021574 | F506F | 55994090 | 0.001 | 0.46 | 0.0 | 0.005 | 0.001 | 0.22 | 0.001 | 0.34 | 0.001 | 0.47 | 0.001 | 0.18 | 0.06 | 0.95 | +?+-+? |
| rs78991146 | A614V | 55994413 | 0.003 | 0.60 | 0.001 | 0.032 | 0.001 | 0.12 | 0.001 | 0.31 | 0.001 | 0.29 | 0.001 | 0.19 | -0.3 | 0.78 | ++---? |
| rs201469671 | C620C | 55994432 | 0.0 | 0.029 | 0.0 | 0.007 | 0.001 | 0.45 | 0.001 | 0.39 | 0.001 | 0.45 | 0.001 | 0.36 | -0.4 | 0.69 | +--+-? |
| rs200197263 | R638R | 55994486 | 0.0 | 0.005 | 0.0 | 0.099 | 0.0 | 0.020 | 0.0 | 0.036 | 0.002 | 0.79 | 0.0 | 0.15 | 0.5 | 0.62 | ++-++? |
| rs114065657 | V801V | 55994975 | 0.001 | 0.61 | 0.001 | 0.74 | 0.002 | 0.30 | 0.001 | 0.34 | 0.003 | 0.64 | 0.004 | 0.36 | 2.39 | 0.017 | +++++? |
| rs201058799 | R820R | 55995032 | 0.002 | 0.50 | 0.002 | 0.81 | 0.001 | 0.64 | 0.002 | 0.70 | 0.001 | 0.73 | 0.001 | 0.47 | -0.4 | 0.69 | ++-+-- |
| rs200801147 | T827M | 55995052 | 0.0 | 0.0 | 0.0 | 0.0 | 0.0 | 0.050 | 0.0 | 0.005 | 0.0 | 0.51 | 0.0 | 0.065 | -0.5 | 0.64 | ??--?? |
| rs73608718 | E835E | 55995077 | 0.002 | 0.81 | 0.0 | 0.22 | 0.003 | 0.24 | 0.001 | 0.32 | 0.002 | 0.55 | 0.006 | 0.44 | 0.67 | 0.50 | -?+-++ |
| rs138326099 | V847V | 55995113 | 0.005 | 0.42 | 0.004 | 0.36 | 0.004 | 0.45 | 0.004 | 0.38 | 0.003 | 0.34 | 0.008 | 0.43 | 0.1 | 0.92 | ++-+-+ |
| rs112775775 | T859S | 55995148 | 0.0 | 0.094 | 0.0 | 0.066 | 0.002 | 0.32 | 0.001 | 0.29 | 0.002 | 0.71 | 0.004 | 0.45 | 1.7 | 0.096 | +-++++ |
| rs150319373 | T860A | 55995150 | 0.005 | 0.72 | 0.002 | 0.63 | 0.004 | 0.44 | 0.002 | 0.49 | 0.003 | 0.52 | 0.002 | 0.36 | -0.4 | 0.72 | +++--? |
| rs73608719 | E909D | 55995299 | 0.002 | 0.81 | 0.0 | 0.22 | 0.003 | 0.24 | 0.001 | 0.32 | 0.002 | 0.55 | 0.006 | 0.44 | 0.7 | 0.51 | -?+-++ |
| rs200160847 | D939V | 55995388 | 0.001 | 0.83 | 0.001 | 0.32 | 0.004 | 0.34 | 0.001 | 0.47 | 0.0 | 0.46 | 0.001 | 0.17 | -0.2 | 0.83 | ?-+--? |
| rs112407198 | T943T | 55995401 | 0.011 | 0.78 | 0.009 | 0.82 | 0.011 | 0.47 | 0.012 | 0.72 | 0.013 | 0.74 | 0.011 | 0.41 | 2.9 | 0.0043 | +-++++ |
| rs149561076 | V947V | 55995413 | 0.001 | 0.15 | 0.0 | 0.044 | 0.001 | 0.40 | 0.002 | 0.45 | 0.002 | 0.60 | 0.001 | 0.39 | 1.0 | 0.30 | -++-+? |
| rs201209790 | G1010G | 55995602 | 0.002 | 0.59 | 0.002 | 0.43 | 0.001 | 0.42 | 0.001 | 0.49 | 0.001 | 0.50 | 0.001 | 0.33 | -0.6 | 0.58 | +-+--? |
| rs73057174 | L1046L | 55995710 | 0.029 | 0.91 | 0.031 | 0.95 | 0.022 | 0.67 | 0.022 | 0.90 | 0.024 | 0.90 | 0.019 | 0.55 | 4.2 | 2.8×10^–5^ | +-++++ |
| rs149742958 | P1047P | 55995713 | 0.0 | 0.0 | 0.0 | 0.0 | 0.001 | 0.84 | 0.0 | 0.003 | 0.0 | 0.65 | 0.0 | 0.077 | 0.05 | 0.96 | ??+--? |

We selected SNPs for each according to their MAF and INFO.

Key: MAF, minor allele frequency; INFO, imputation quality score made by IMPUTE2

^*^ The number of SNPs used in each study

^§^ The signs mean those of the *Z* score of each study. The question mark means missing data in the study because of low INFO or high MAF. The order of the signs is ADNI, ADNI2, GenADA, eMERGE, and NIA, Framingham study

**Table S3. Significant principal components (PC) and variance inflation factors (VIF) between *ZNF628* and *APOE* ɛ4**

| **Study** | **PC** | **VIF** |
| --- | --- | --- |
| **ADNI** | 3 | 1.00519 |
| **ADNI2** | 9 | 1.00007 |
| **GenADA** | 2 | 1.00000 |
| **eMERGE** | 6 | 1.00051 |
| **NIA-LOAD** | None | 1.00005 |
| **Framingham** | None | 1.00000 |

**References**

[1] McKhann G, Drachman D, Folstein M, Katzman R, Price D, Stadlan EM (1984) Clinical diagnosis of Alzheimer's disease: report of the NINCDS-ADRDA Work Group under the auspices of Department of Health and Human Services Task Force on Alzheimer's Disease. *Neurology* **34**, 939-944.

[2] Gearing M, Mirra SS, Hedreen JC, Sumi SM, Hansen LA, Heyman A (1995) The Consortium to Establish a Registry for Alzheimer's Disease (CERAD). Part X. Neuropathology confirmation of the clinical diagnosis of Alzheimer's disease. *Neurology* **45**, 461-466.

[3] Braak H, Braak E (1991) Neuropathological stageing of Alzheimer-related changes. *Acta Neuropathol* **82**, 239-259.

[4] Khachaturian ZS (1985) Diagnosis of Alzheimer's disease. *Arch Neurol* **42**, 1097-1105.

[5] Hyman BT, Trojanowski JQ (1997) Consensus recommendations for the postmortem diagnosis of Alzheimer disease from the National Institute on Aging and the Reagan Institute Working Group on diagnostic criteria for the neuropathological assessment of Alzheimer disease. *J Neuropathol Exp Neurol* **56**, 1095-1097.

[6] Aulchenko YS, Ripke S, Isaacs A, van Duijn CM (2007) GenABEL: an R library for genome-wide association analysis. *Bioinformatics* **23**, 1294-1296.

[7] Novembre J, Stephens M (2008) Interpreting principal component analyses of spatial population genetic variation. *Nat Genet* **40**, 646-649.

[8] Aulchenko YS, Struchalin MV, van Duijn CM (2010) ProbABEL package for genome-wide association analysis of imputed data. *BMC Bioinformatics* **11**, 134.

[9] Bertram L, Tanzi RE (2008) Thirty years of Alzheimer's disease genetics: the implications of systematic meta-analyses. *Nat Rev Neurosci* **9**, 768-778.

[10] Giardine B, Riemer C, Hardison RC, Burhans R, Elnitski L, Shah P, Zhang Y, Blankenberg D, Albert I, Taylor J, Miller W, Kent WJ, Nekrutenko A (2005) Galaxy: a platform for interactive large-scale genome analysis. *Genome Res* **15**, 1451-1455.
